# Persistent Median Arteries: A Cadaveric Study of Prevalence, Variants, and Clinical Relevance

**DOI:** 10.7759/cureus.101320

**Published:** 2026-01-11

**Authors:** Suryakanta Seth, Sarah S Sangma, Chetan Sahni, Abdul Kareem, Abhimanyu Vasudeva

**Affiliations:** 1 Anatomy, All India Institute of Medical Sciences, Gorakhpur, Gorakhpur, IND; 2 Physical Medicine and Rehabilitation, All India Institute of Medical Sciences, Gorakhpur, Gorakhpur, IND

**Keywords:** anatomical variation, cadaveric study, carpal tunnel syndrome, median nerve, persistent median artery

## Abstract

The median artery, an embryonic vessel, may persist into adulthood as the persistent median artery (PMA), a variant of clinical and surgical relevance. In a cadaveric study of 23 upper limbs from 12 donated adult cadavers, PMA was identified in eight limbs (34.8%). Half of these (4/8 limbs, 17.4%) pierced the median nerve in the proximal forearm, an uncommon finding. Two palmar-type PMAs were identified, both of which exhibited atypical terminal branching in the hand (2/23 limbs, 8.7%). These anatomical variations may contribute to median nerve compression syndromes, such as carpal tunnel syndrome, and pose risks during surgical procedures of the forearm and wrist. Awareness of PMA anatomy, including its prevalence and variant patterns, is important for accurate diagnosis and safe surgical planning.

## Introduction

During early development, the median artery serves as the primary blood supply to the forearm and hand. However, it typically regresses after the eighth week as the radial and ulnar arteries develop. When this regression does not occur, the artery can persist into adulthood as a variant known as the persistent median artery (PMA), often accompanying the median nerve [1,2]. Anatomical studies and meta-analyses indicate that the PMA varies in prevalence and course among populations and may contribute to the superficial palmar arch (SPA) [1,3,4]. The PMA is commonly classified as either an antebrachial type, terminating in the forearm, or a palmar type, extending into the hand. In some cases, it is associated with variations of the median nerve, such as a high division before entering the carpal tunnel. Clinically, the presence of a PMA can contribute to neurovascular compression syndromes, including carpal tunnel syndrome, pronator syndrome, or anterior interosseous nerve compression. Awareness of these variations is important for clinicians and surgeons, as the PMA may influence surgical approaches, vascular procedures, and the management of nerve-related conditions in the forearm, wrist, and hand [1-4]. Studies indicate that the prevalence of the median artery in adults has increased over time, possibly reflecting microevolutionary changes, with recent data showing it in over 30% of individuals. This trend suggests that the median artery may no longer be considered a rare anatomical variant in future populations, emphasizing the need for continued awareness in clinical and surgical practice [5]. This study aims to determine the prevalence of the PMA, characterize its anatomical variants, including rare piercing forms, and assess their potential clinical relevance in the forearm and hand.

## Materials and methods

Study design

This was a descriptive cadaveric anatomical study conducted in the department of anatomy of a tertiary care hospital and its attached medical college in Northern India. The study was observational in nature and involved systematic dissection of upper limbs from a consecutive cohort of cadavers during routine undergraduate anatomy teaching over a three-year period from September 2022 to September 2025. The study material consisted of 23 upper limbs from 12 donated adult cadavers, with an age range of 50-60 years. Of these, 12 limbs were right-sided, and 11 were left-sided, ensuring representation from both sides, while one additional left limb was excluded due to damaged vascular anatomy. Limb dominance was recorded where possible, but the biological sex of the donors was not formally recorded and therefore was not included as a study variable. Exact ages were not available for all specimens.

Inclusion and exclusion criteria

All adult cadavers available through the institutional Voluntary Body Donation Program during the study period were considered for inclusion. Cadavers were included if their upper limb neurovascular anatomy was intact. Cadavers with evidence of prior upper limb surgery, trauma, or gross pathology affecting the forearm or hand were excluded. Specimens with damaged vascular anatomy that prevented accurate assessment of the PMA were also excluded.

Ethical statement

This study was conducted on bodies donated through the institutional Voluntary Body Donation Program. In accordance with institutional and national guidelines, ethical clearance from the Institutional Ethics Committee was not required for research on donated cadavers. All cadavers were received with written informed consent from the donors or their legal heirs. All procedures complied with institutional norms for cadaver use, and the manuscript was prepared in accordance with the principles of the Declaration of Helsinki.

Dissection procedure and study measures

Dissections were performed following standard anatomical protocols, with systematic exposure of the neurovascular structures from the cubital fossa to the hand. Particular attention was directed toward identifying the PMA. Differentiation of the PMA from other vessels was achieved through meticulous anatomical dissection based on its origin, course, and terminal branching pattern; no vascular injection or staining techniques were employed. Each specimen was evaluated for the presence or absence of the PMA, its site of origin, course in the forearm, and relationship to the median nerve, including documentation of piercing or non-piercing patterns, passage through the carpal tunnel, and termination in the hand. Observations were recorded contemporaneously during dissection. As this was a direct cadaveric study, blinding was not applicable.

Based on its course and termination, the PMA was identified as either an antebrachial pattern, in which the artery accompanies the median nerve in the forearm and terminates proximally without entering the carpal tunnel or contributing to the SPA, or a palmar pattern, in which the artery accompanies the median nerve through the carpal tunnel and contributes partially or completely to the SPA, as described previously [1].

Statistical analysis

Data were analyzed descriptively. The number of upper limbs exhibiting each anatomical pattern of the PMA was recorded, and results were expressed as counts and percentages. No inferential statistical tests were performed. To enhance interpretability and reproducibility, a summary table detailing PMA types, laterality, number of limbs, median nerve piercing, and terminal branching patterns was prepared, providing a clear overview of anatomical variations observed.

## Results

A PMA was identified in eight of the 23 dissected upper limbs (34.8%), with equal representation on the left and right upper limbs (four left, four right; 50% each). No bilateral occurrence of the PMA was observed. In the remaining 15 limbs (65.2%), the median artery was absent, and the arterial anatomy followed the usual pattern without additional vascular variations.

**Figure 1:**
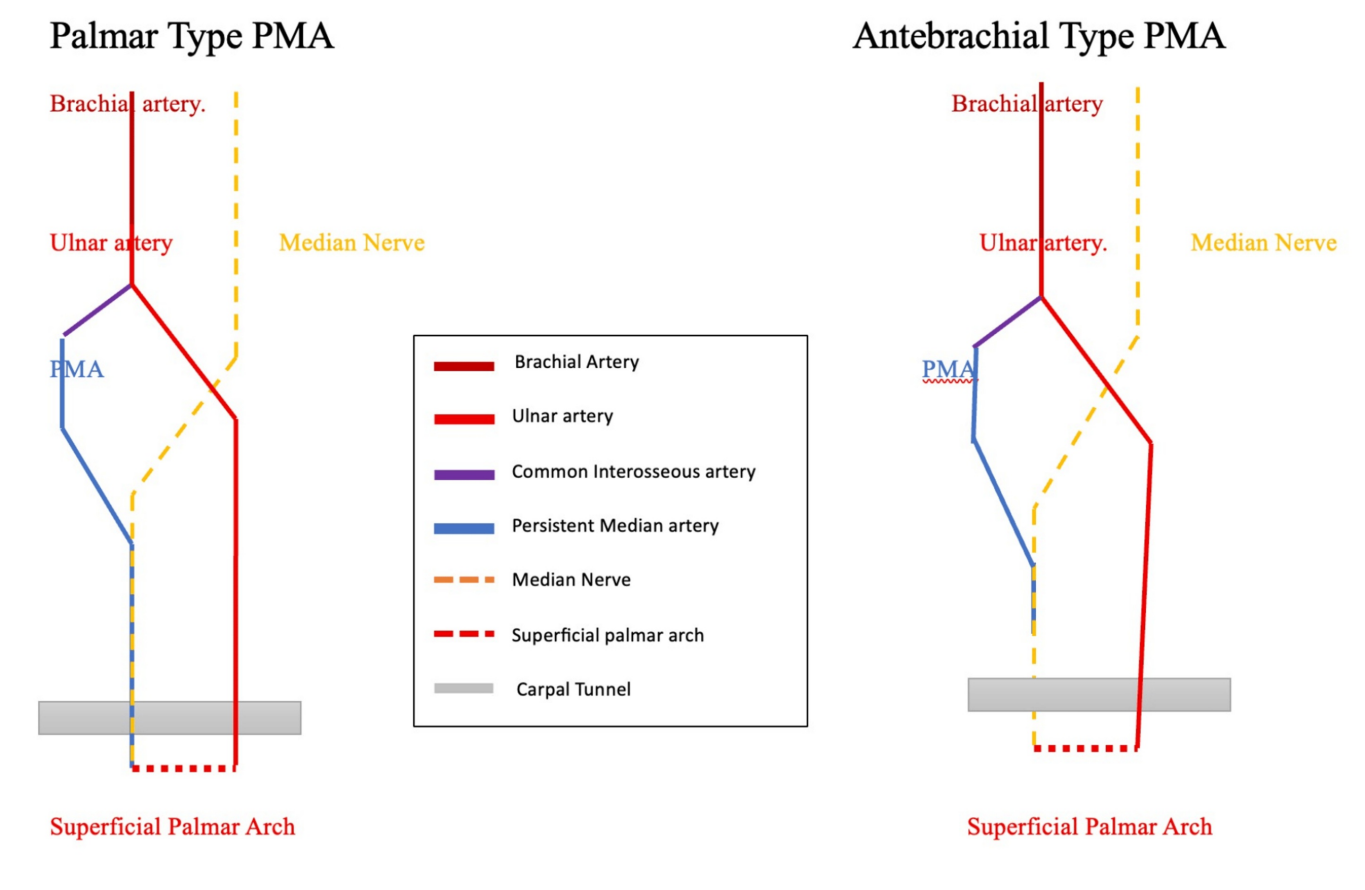
Schematic illustration of antebrachial and palmar types of PMA PMA: persistent median artery

Among the limbs exhibiting a PMA, six arteries were of the antebrachial type, while two arteries were classified as the palmar type [1]. The antebrachial and palmar patterns of the PMA observed in the present study are illustrated schematically in Figure 1.

**Figure 2:**
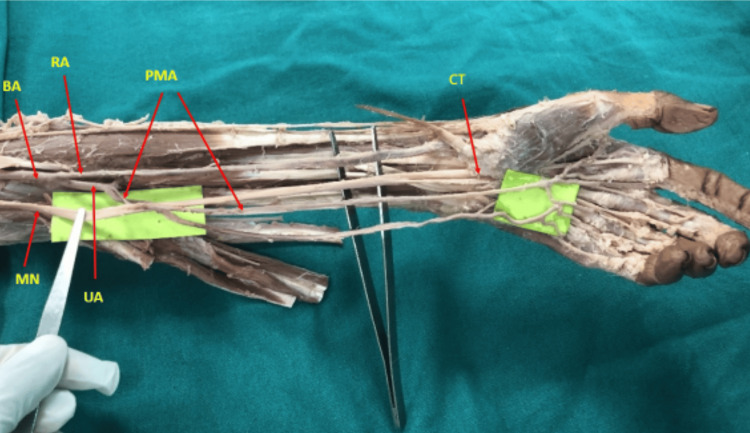
PMA piercing the median nerve in the proximal forearm (left upper limb). The artery is seen coursing through the nerve, highlighted with arrowheads BA: brachial artery, UA: ulnar artery, RA: radial artery, MN: median nerve, PMA: persistent median artery, CT: carpal tunnel

The PMA originated from the ulnar artery in three limbs, from the common interosseous artery in four limbs, and from the anterior interosseous artery in one limb. In four cases (50% of PMA variants), the artery pierced the median nerve in the proximal third of the forearm (Figure 2).

**Figure 3:**
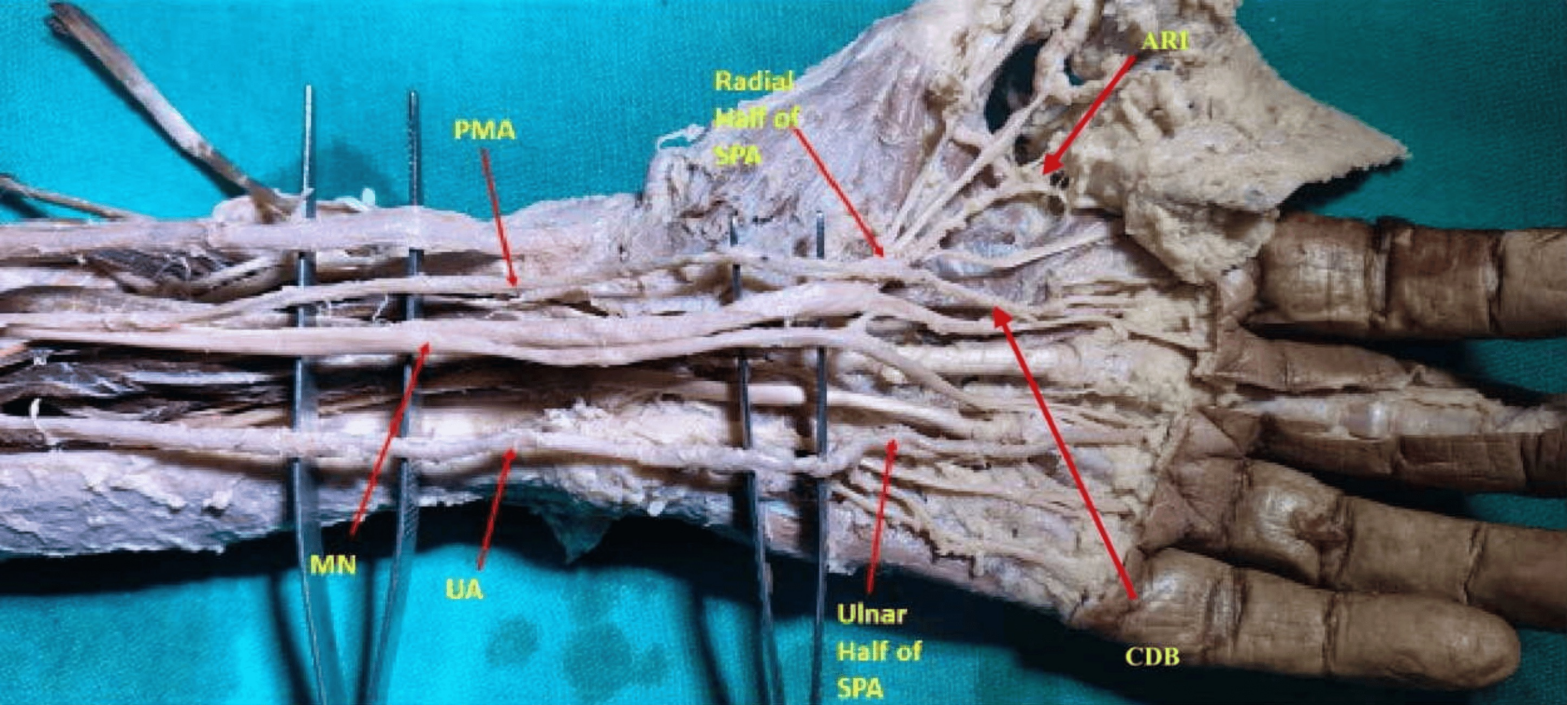
Palmar-type PMA forming a radial incomplete SPA (left upper limb). Arrow indicates the PMA supplying the thumb and index finger MN: median nerve, UA: ulnar artery, PMA: persistent median artery, SPA: superficial palmar arch, ARI: arteria radialis indices, CDB: common digital branch

Two antebrachial-type PMAs terminated within the nerve’s perineurium, while two palmar-type PMAs exhibited distinct terminal branching patterns in the hand. In one left upper limb, a palmar-type PMA originating from the ulnar artery pierced the median nerve, traversed the carpal tunnel, and formed the radial portion of an incomplete SPA. This vessel supplied the thumb, index finger, and a common digital branch, and also provided an anastomotic connection to the radial artery without connection to the ulnar artery (Figure 3).

**Figure 4:**
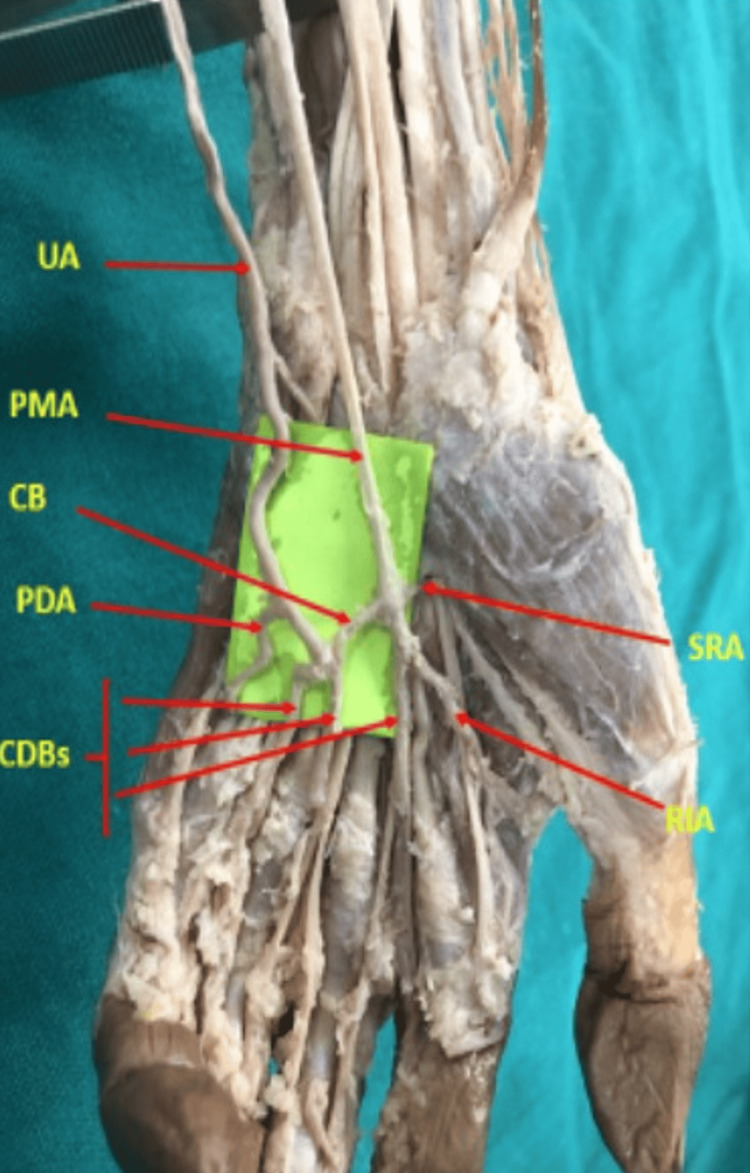
Palmar-type PMA completing the SPA via a thin communicating branch (left upper limb). Note the branch connecting to the ulnar artery and also giving rise to radialis indicis artery and a common digital branch UA: ulnar artery, PMA: persistent median artery, PDA: proper digital artery, RIA: radial indicis artery, CB: communicating branch, CDBs: common digital branches, SRA: superficial radial artery

In another left upper limb, a palmar-type PMA arising from the common interosseous artery pierced the median nerve, passed through the carpal tunnel, and completed the SPA via a thin communicating branch to the ulnar artery. This vessel also gave rise to the radialis indicis artery and a common digital branch (Figure 4).

**Figure 5:**
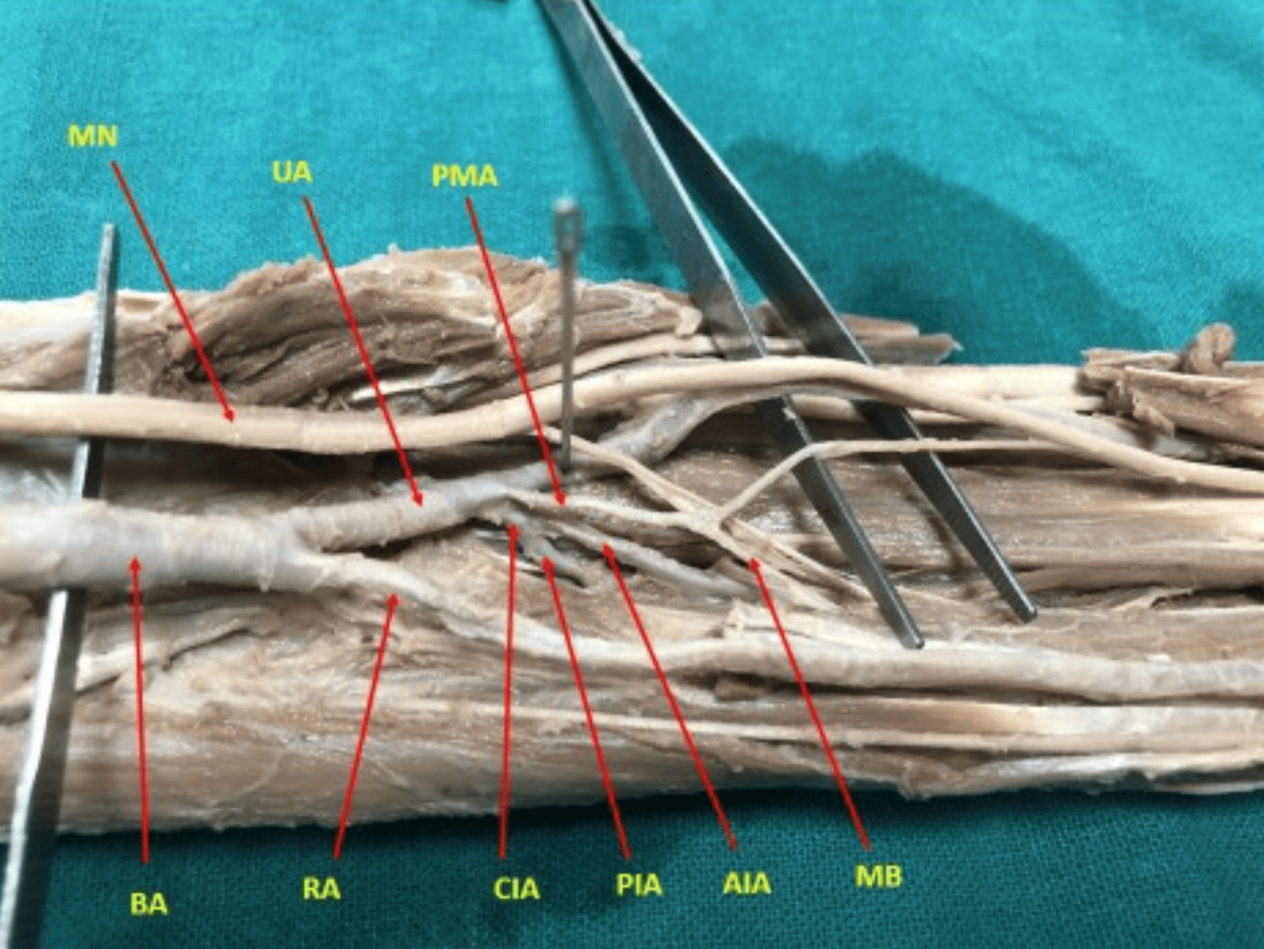
Antebrachial type of PMA blending with the perineurium of the median nerve in the proximal forearm BA: brachial artery, UA: ulnar artery, RA: radial artery, MN: median nerve, PMA: persistent median artery, CIA: common interosseous artery, AIA: anterior iterosseous artery, PIA: posterior interosseous artery, MB: muscular branch, CT: carpal tunnel

The remaining antebrachial-type PMAs terminated in the forearm without entering the carpal tunnel (Figure 5).

To summarize, the PMA was identified in eight specimens, with equal right- and left-sided distributions (four each), indicating no laterality predominance. The PMA most frequently originated from the common interosseous artery (four cases), followed by the ulnar artery (three cases), with a single case arising from the anterior interosseous artery. Piercing of the median nerve was observed in four cases (50%), while passage through the carpal tunnel was uncommon and noted in only two specimens. Contribution to the SPA was rare, with one case demonstrating partial and one case demonstrating complete contribution. In contrast, in the remaining six cases, the PMA terminated within the median nerve (antebrachial type) (Table 1).

**Table 1:** Summary of anatomical variations of PMA PMA: persistent median artery, SPA: superficial palmar arch

Serial number	PMA type	Laterality	Origin	Pierces the median nerve	Termination
1	Antebrachial type	Right	Ulnar artery	No	Terminates in the forearm by blending with the median nerve in the upper third of the forearm.
2	Antebrachial type	Right	Common interosseous artery	No	Terminates in the forearm by blending with the median nerve in the upper third of the forearm.
3	Antebrachial type	Right	Common interosseous artery	No	Terminates in the forearm by blending with the median nerve in the upper third of the forearm.
4	Antebrachial type	Left	Anterior interosseous artery	No	Terminates in the forearm by blending with the median nerve in the middle third of the forearm.
5	Antebrachial type	Right	Ulnar artery	Yes	Terminates in the forearm by blending with the median nerve in the middle third of the forearm.
6	Palmar type	Left	Common interosseous artery	Yes	Forms the radial half of SPA and gives rise to an incomplete arch.
7	Antebrachial type	Left	Ulnar artery	Yes	Terminates in the forearm by blending with the median nerve at the lower third of the forearm.
8	Palmar type	Left	Common interosseous artery	Yes	Forms the radial half of SPA and completes the SPA by joining with the ulnar half of SPA by a short, thin communicating branch.

## Discussion

Building on its embryologic origins, PMA represents a recognized vascular variant rather than a pathologic finding. This embryology-based anomaly may influence median nerve vascularity and contribute variably to palmar arch formation. Such heterogeneity provides a framework for understanding reported differences in prevalence and anatomical patterns across populations [2,3].

Half of the identified PMAs pierced the median nerve in the proximal forearm. Previous large-scale analyses have highlighted substantial variability in reported PMA prevalence, with the antebrachial type being more common than the palmar type. Meta-analytic data indicate that while both forms are relatively frequent in cadaveric studies, the palmar variant is observed less often in patients undergoing carpal tunnel surgery, suggesting that its direct contribution to median nerve compression may be limited. Nevertheless, both anatomical patterns remain clinically relevant, emphasizing the importance of awareness during forearm and carpal tunnel procedures [6].

The clinical significance of the PMA is twofold. First, as a space-occupying structure within the rigid confines of the carpal tunnel, a patent PMA can contribute to median nerve compression and carpal tunnel syndrome [7,8]. Thrombosis of a PMA within the tunnel is a recognized, though rare, cause of acute compressive neuropathy [9]. Second, from a surgical perspective, an unrecognized PMA poses a risk of iatrogenic injury and significant hemorrhage during procedures in the distal forearm, wrist, or hand, particularly following tourniquet release [10]. The palmar-type PMA can be a major source of blood supply to the thumb and radial digits, as seen in our cases; its inadvertent ligation could lead to digital ischemia.

The piercing variant described here may have further implications. A nerve penetrated by an artery may represent a site of inherent structural weakness, potentially predisposing to focal neuropathy, especially in conditions like diabetes mellitus [11]. For surgeons performing procedures such as carpal tunnel release, forearm fasciotomy, or nerve decompression, preoperative awareness of such variations is crucial. Imaging with high-resolution ultrasound or MRI can reliably identify a PMA and define its relationship to the median nerve [12]. Similar observations have been reported in the literature, where a PMA pierced the median nerve, traversed the carpal tunnel, and contributed to the SPA, supplying multiple digital branches, including the radialis indicis and princeps pollicis. These findings underscore the clinical relevance of such variants for surgical planning and management of hand disorders [13].

Our observation of a PMA communicating with the ulnar digital arteries illustrates the PMA's potential to form atypical collateral pathways in the hand. This reinforces the principle that hand vascular anatomy is highly variable, and assumptions based on textbook patterns are insufficient for precise surgical planning. Anatomical analyses of the SPA have demonstrated considerable variability in arch morphology and branching patterns, including short or incomplete arches. Accessory vessels, such as a PMA, may provide important collateral supply in such hands, particularly when the palmar arch is incomplete. Recognition of these variations is essential for surgical procedures involving microvascular reconstruction, hand surgery, or radial artery harvesting, as incomplete arches or atypical communicating branches may affect perfusion and increase the risk of ischemic complications [14].

In this series, based on dissections conducted from September 2022 to 2025, the prevalence of PMA was 34.8%, consistent with higher estimates from recent studies, which suggest a possible microevolutionary increase in this variant (Table 2) [5].

**Table 2:** Summary of published studies reporting the prevalence of PMA PMA: persistent median artery

Serial number	Author (year)	Prevalence, %
1.	Rodriguez-Niedenführ et al. (1999) [3]	12.00
2.	Natsis et al. (2009) [2]	2.78
3.	Singla et al. (2012) [4]	6.67
4.	Chen et al. (2017) [12]	7.50
5.	Haładaj et al. (2019) [7]	4.00
6.	Lucas et al. (2020) [5]	33.33
7.	Simić et al. (2024) [14]	5.00
8.	Ellis et al. (2025) [1]	43.00
9.	Seth et al. (2026) (present study)	34.8

This study has certain limitations inherent to its design. The sample size was limited and derived from a single tertiary care hospital and its attached medical college, which may restrict the generalizability of the findings to broader populations. As a cadaveric study, the observations reflect anatomical presence and variation but do not provide information regarding vascular patency, hemodynamics, or direct clinical correlations. Additionally, the age range of the cadavers was limited to older adults, and variations in younger populations could not be assessed. Despite these limitations, the study provides valuable anatomical insights into the prevalence and patterns of the PMA, including rare piercing and palmar variants of surgical relevance.

We emphasize that larger and more diverse populations, as well as prospective imaging and functional studies in living subjects, could clarify the clinical significance of PMA variations. Additionally, quantitative measurements of PMA morphology and branching patterns, and their correlation with surgical outcomes, may further support preoperative planning and improve patient safety.

## Conclusions

The PMA is a common anatomical variant. Variations, including rare forms that pierce the median nerve or contribute significantly to hand circulation, have direct clinical implications for the diagnosis of neuropathies and the safety of upper limb surgery. Recent evidence suggests that the prevalence of the PMA may be increasing, highlighting the importance of continued awareness among clinicians and surgeons. Understanding these variations is essential for accurate diagnosis, safe surgical planning, and optimizing patient outcomes. Future studies with larger, diverse populations are warranted to further explore the anatomical and clinical significance of the PMA.
